# Reverse order law for outer inverses and Moore-Penrose inverse in the context of star order

**DOI:** 10.12688/f1000research.123411.1

**Published:** 2022-07-27

**Authors:** Umashankara Kelathaya, Manjunatha Prasad Karantha

**Affiliations:** 1 Department of Data Science, Prasanna School of Public Health, Manipal Academy of Higher Education, Manipal, Karnataka, 576104, India

**Keywords:** Generalized inverses, Outer inverses, Moore-Penrose inverse, Reverse order law, Star order

## Abstract

The reverse order law for outer inverses and the Moore-Penrose inverse is discussed in the context of associative rings. A class of pairs of outer inverses that satisfy reverse order law is determined. The notions of left-star and right-star orders have been extended to the case of arbitrary associative rings with involution and many of their interesting properties are explored. The distinct behavior of projectors in association with the star, right-star, and left-star partial orders led to several equivalent conditions for the reverse order law for the Moore-Penrose inverse.

## Introduction

Given any invertible matrices *A* and *B*, it is well-known that ( *AB*) ^−1^ = *B* ^−1^ *A* ^−1^. This property is often called *reverse order law* for the invertible matrices. This law is easily extended for the invertible elements from an associative ring with identity. This notion has been well studied in the literature and it is known that the analogue is not true in the case of generalized inverses, unless there are certain additional conditions. Several researchers came up with many necessary as well as sufficient conditions for the reverse order law to hold in the case of different generalized inverses. Ivan Erdelyi in 1966 discussed reverse order law for Moore-Penrose inverses of complex matrices in the context of partial isometries ^1^. Greville, in his celebrated paper ^2^, gave several equivalent conditions for the reverse order law for Moore-Penrose inverse of matrices over complex field. He proved that given two complex matrices *A* and *B* with *AB* defined, the reverse order law ( *AB*) ^†^ = *B* ^†^ *A* ^†^ holds if and only if the equations *A* ^†^ ABB* A* = BB* A* and *BB* ^†^ A* *AB* = A* *AB* are satisfied, which is equivalent to the column space inclusions ( BB* A*) ⊆ ( A*) and ( A* *AB*) ⊆ ( *B*). Here, the notations ^†^ and * denote the Moore-Penrose inverse and conjugate transpose, respectively. Motivated by this paper, Hartwig discussed equivalent conditions for triple reverse order law for Moore-Penrose inverses (see, 3) and Y. Tian discussed equivalent conditions for multiple reverse order law for Moore-Penrose inverses of complex matrices (see, 4).

The reverse order law for Moore-Penrose inverses in the context of elements from an associative ring with involution was studied previously ^5^. Also, it has been extended to different generalized inverses including group inverse, Drazin inverse, core inverse, Drazin Moore-Penrose (DMP) inverse and pseudo core inverse, of matrices over a field, operators on Hilbert space, and elements of rings. Interested readers may refer to 4, 6– 10 and the references therein.

Another notion of our concern is the star order, introduced by M. P. Drazin in 1978 ^11^. In the beginning of 90’s, J. K. Baksalary and S. K. Mitra considered weaker conditions than those considered by Drazin and introduced left-star and right-star orders ^12^. Benitez *et al.*, ^13^ discussed some equivalent conditions for reverse order law for group inverse and Moore-Penrose inverse in terms of sharp and star partial orders, respectively, where most of his results assume that one of the matrices under consideration is Equal Projector (EP).

In the present paper, one of our main focuses is to study the reverse order law for Moore-Penrose inverses of arbitrary elements, which need not be EP, in terms of different partial orders. While exploring the extension of reverse order law for the class of outer inverses, it may be noted that an outer inverse of given element is not uniquely established, unlike in the cases of inverse, Moore-Penrose inverse and group inverses. So, a natural question arises is that whether every outer inverse ( *ab*) ^=^ of *ab* could be written as *g _b_g _a_* for some outer inverses *g _b_* of *b* and *g _a_* of *a*. In case the answer is affirmative, then verify if


$$\left\{{\left(ab\right)}^{=}\}=\{b^{=}a^{=}\}\;(\text{reverse}\;\text{order}\;\text{law?}\right)$$

holds. While answering these questions, a few noteworthy properties of right-star, left-star and star partial orders are studied. Further, we give some equivalent conditions for the reverse order law for Moore-Penrose inverse in terms of different star partial orders.

## Preliminaries

Throughout our discussion, denotes an associative ring, need not be with unity unless indicated otherwise. We use the notations *a*, *b*, *c*, ... for the elements of a ring and *A*, *B*, *C*, ... for matrices under discussion. Let *a* ∈ . If there exists a *g* ∈ satisfying *aga* = *a*, then *a* is said to be *regular* and *g* is called a *generalized inverse* (or simply a *g-inverse*) of *a*. An arbitrary g-inverse of *a* is denoted by *a ^−^*. If there exists a *g* ∈ satisfying *gag* = *g*, then such a *g* is called an *outer inverse* of *a*. An arbitrary outer inverse of *a* is denoted by *a* ^=^. A generalized inverse, which is also an outer inverse, is called a *reflexive generalized inverse* and an arbitrary reflexive generalized inverse of *a* is denoted by $a_{r}^{-}$ . A commuting reflexive generalized inverse of *a* is called the group inverse of *a* and it is denoted by *a* ^#^. It is unique whenever it exists. The classes { *a* ^−^}, { *a* ^=^}, and { $a_{r}^{-}$ } denote the class of all g-inverses, outer inverses and reflexive g-inverses of *a*, respectively. For basic notions of generalized inverses in the context of matrices we refer the readers to 14, 15, and 16.

An involution ‘*’ of a ring is an anti-automorphism whose square is identity; *i.e.*


$$(a^{*}{)}^{*}=a\;{\left(a+b\right)}^{*}=a^{*}+b^{*}\;{\left(ab\right)}^{*}=b^{*}a^{*}\;\text{for}\;\text{all}\;a,b\in .$$

Involution ‘*’ is said to be *proper* (see 11) if for all *a*, *b* ∈ ,


$$a^{*}a=a^{*}b=b^{*}a=b^{*}b\Rightarrow a=b.$$

A *proper* *- *ring* is a ring equipped with a proper involution ‘*’. A ring is said to be *regular ring* if all of its elements are regular. A *- *regular ring* is a regular ring with involution ‘*’ such that a* *a* = 0 ⇒ *a* = 0. Observe that the involution is proper if and only if a* *a* = 0 ⇒ *a* = 0 holds. An important consequence of this property of involution is the *- *cancellation law*.

An element *a* is said to satisfy left (right) *-cancellation law if a* *ax* = a* *ay* ⇒ *ax* = *ay* ( xaa* = yaa* ⇒ *xa* = *ya*). Element *a* is said to be *-cancellable if it satisfies both left and right *-cancellation laws.

For an associative ring with involution '*' and *a* ∈ , consider the following four equations:


axa=a(1)

xax=x(2)

$${\left(ax\right)}^{*}=ax\;(3)$$

$$\;{\left(xa\right)}^{*}=xa.\;(4)$$

An element *g* satisfying the equations { *i*, *j*, ...} ⊆ {1, 2, 3, 4}, is called { *i*, *j*, ...}-inverse of *a* and the class of all { *i*, *j*, ...}-inverses of *a* is denoted by { *a* ^{ *i*, *j*,...}^}. An element *g* ∈ satisfying all of (1), (2), (3) and (4), if exists, is unique and is called the *Moore-Penrose inverse* of *a*. The existence of Moore-Penrose inverse for the elements of a *-regular ring was proved by Kaplansky ^17^, almost at the same time that Penrose ^18^ proved the existence in the case of matrices. The Moore-Penrose inverse of an element *a* is denoted by *a* ^†^. We will use the notation ^†^ to denote the set of all elements of having Moore-Penrose inverse. Note that the elements of ^†^ are *-cancellable.

An element *a* is *Hermitian* if a* = *a*, and it is an *idempotent* if *a* ^2^ = *a*. Hermitian idempotents are called *projectors*. If *a* ^†^ is the Moore-Penrose inverse of *a*, then both *aa* ^†^ and *a* ^†^ *a* are projectors.

The right annihilator *a ^o^* of an element *a* ∈ is the set { *x* ∈ : *ax* = 0} and the left annihilator *^o^a* is the set { *y* ∈ : *ya* = 0}. If 1 ∉ , then the right annihilator (1 − *a*) *^o^* is defined as { *x* ∈ : *x* = *ax*} and the left annihilator *^o^* (1 − *a*) = { *y* ∈ : *y* = *ya*} (see 19). For any *a*, *b* ∈ , we write $a\underset{cs}{≃}b$ ( $a\underset{rs}{≃}b$ ) if the principal right (left) ideals generated by *a* and *b* coincide. We say that *a* and *b* are space equivalent if both $a\underset{cs}{≃}b$ and $a\underset{rs}{≃}b$ are true. In such a case we write $a\underset{sp}{≃}b$ .

The sum *a* + *b* is written as *a* ⊕ *b* (⊕ refers to direct sum) if *a* ∩ *b* = {0} and *a* ∩ *b* = {0}. If so is the case, then we say that *a* and *b* are disjoint. We refer to 19, 20, and 21 for more details on the above notions.

The minus partial order is helpful in discussing many of the properties of star partial order. For the initial works on minus partial order, one may refer to 21– 23.

**Definition 1** (Minus Partial Order ^21, 22^). *The minus partial order is a relation, denoted by* ≤ ^−^, *defined on set of all regular elements of by a* ≤ ^−^ *b if there exists a g-inverse g of a such that ag* = *bg and ga* = *gb*.

For the proof of the following theorem, see 24 and 25.

**Theorem 2** (Right-left Symmetry ^24, 25^). *Let be an associative ring with unity and let a*, *b*, *c* ∈ *such that a* = *b* + *c is regular. Then the following statements are equivalent.*

(i) *b* ≤ ^−^ *a*
(ii) *b* ⊕ *c* = *a*
(iii) *b* ∩ *c* = {0} = *b* ∩ *c*
(iv) *b* ⊕ *c* = *a*
(v) *c* ≤ ^−^ *a*
(vi) *b* ∈ *a* ∩ *a and* { *a* ^−^} ⊆ { *b* ^−^}
(vii) *c* ∈ *a* ∩ *a and* { *a* ^−^} ⊆ { *c* ^−^}.

Note that the theorem holds even in the case of an associative ring without unity, provided *a*, *b*, *c* are regular.

**Definition 3** (Star Order ^11^). Let be an associative ring with involution ‘* *’ and a*, *b* ∈ . *The relation* ≤* *defined by a* ≤* *b* if a* *a* = a* b and aa* = ba* *is called star order on *. *If a* ≤* *b*, *then we say that the element a is dominated by b under star order*.

Drazin, in 11 noticed that the relation star order defined on a *-semigroup is a partial order. He pointed out that if the Moore-Penrose inverses of *a* and *b* exist, then,


$$a\leq^{*}b\Leftrightarrow a^{†}a=a^{†}b\;\text{and}\;ba^{†}=aa^{†}\;(5)$$

and


$$a\leq^{*}b\Leftrightarrow a^{†}a=b^{†}a\;\text{and}\;aa^{†}=ab^{†}\text{.}\;(6)$$

He also noted that


$$a\leq^{*}b\Leftrightarrow a^{†}\leq^{*}b^{†}\text{.}\;(7)$$

**Definition 4.** *A partial order* ≤ _2_ *on a set S*_2_ *is said to be dominated by a partial order* ≤ _1_ *defined on set S*_1_ *if S*_2_ ⊆ *S*_1_ *and for a*, *b* ∈ *S*_2_,


$$b\leq_{2}a\Rightarrow b\leq_{1}a.$$

Note that the minus partial order dominates the star partial order (as seen from ( 5)).

## Reverse order law for outer inverses

In this section we probe if the reverse order law mentioned in the earlier section holds. In the following lemma, we observe that the inclusion {( *ab*) ^=^} ⊆ { *b* ^=^ *a* ^=^} always holds.

**Lemma 5.** *For a*, *b* ∈ , *we have*


$$\{{\left(ab\right)}^{=}\}\subseteq \{b^{=}a^{=}\}.\;(8)$$

*Proof.* Given an outer inverse *g _ab_* of *ab*, note that *g _ab_a* is an outer inverse of *b* and *bg _ab_* is an outer inverse of *a*. Further, we have *g _ab_* = *g _ab_* ( *ab*) *g _ab_* = ( *g _ab_a*)( *bg _ab_*) ∈ { *b* ^=^ *a* ^=^}, proving ( 8). Hence the lemma.

Unfortunately, the reverse of inclusion appearing in ( 8) need not be true. We have a counter example in the matrix case. For $A=\left[\begin{array}{l} \begin{array}{l} 1\\ 2 \end{array} \end{array}\;\begin{array}{l} \begin{array}{l} 3\\ 6 \end{array} \end{array}\right]$ and $B=\left[\begin{array}{l} \begin{array}{l} 2\\ 2 \end{array} \end{array}\;\begin{array}{l} \begin{array}{l} 1\\ 1 \end{array} \end{array}\right]$ , consider $G_{A}=\left[\begin{array}{l} \begin{array}{l} 1\\ 0 \end{array} \end{array}\;\begin{array}{l} \begin{array}{l} 0\\ 0 \end{array} \end{array}\right]$ and $G_{B}=\left[\begin{array}{l} \begin{array}{l} 0\\ 1 \end{array} \end{array}\;\begin{array}{l} \begin{array}{l} 0\\ 0 \end{array} \end{array}\right]$ , which are outer inverses of *A* and *B*, respectively. Note that $G_{B}G_{A}=\left[\begin{array}{l} \begin{array}{l} 0\\ 1 \end{array} \end{array}\;\begin{array}{l} \begin{array}{l} 0\\ 0 \end{array} \end{array}\right]$ is not an outer inverse of $AB=\left[\begin{array}{l} \begin{array}{l} 8\\ 16 \end{array} \end{array}\;\begin{array}{l} \begin{array}{l} 4\\ 8 \end{array} \end{array}\right]$ , as we have 


$$G_{B}G_{A}ABG_{B}G_{A}=\left[\begin{array}{l} \begin{array}{l} 0\\ 1 \end{array} \end{array}\;\begin{array}{l} \begin{array}{l} 0\\ 0 \end{array} \end{array}\right]\;\left[\begin{array}{l} \begin{array}{l} 8\\ 16 \end{array} \end{array}\;\begin{array}{l} \begin{array}{l} 4\\ 8 \end{array} \end{array}\right]\;\left[\begin{array}{l} \begin{array}{l} 0\\ 1 \end{array} \end{array}\;\begin{array}{l} \begin{array}{l} 0\\ 0 \end{array} \end{array}\right]=\left[\begin{array}{l} \begin{array}{l} 0\\ 4 \end{array} \end{array}\;\begin{array}{l} \begin{array}{l} 0\\ 0 \end{array} \end{array}\right]\neq \left[\begin{array}{l} \begin{array}{l} 0\\ 1 \end{array} \end{array}\;\begin{array}{l} \begin{array}{l} 0\\ 0 \end{array} \end{array}\right].$$

This proves that { *B* ^=^ *A* ^=^} ≠ {( *AB*) ^=^}.

In 26 and 27, the authors presented necessary and sufficient conditions for { *B* ^{1,2,3}^ *A* ^{1,2,3}^} ⊆ {( *AB*) ^{1,2,3}^} in the case of matrices and bounded linear operators on Hilbert spaces, respectively. An equivalent condition for the inclusion { *B* ^{1,2}^ *A* ^{1,2}^} ⊆ {( *AB*) ^{1,2}^} is given in 28 for bounded linear operators on complex Hilbert spaces.

Now, our interest in this section is to characterize the subset of { *b* ^=^ *a* ^=^}, which equals to {( *ab*) ^=^}, where *a*, *b* ∈ .

**Theorem 6.** *Let be any associative ring and let a*, *b* ∈ . Then,


$$\{{\left(ab\right)}^{=}\}=\{g_{b}g_{a}\;:\;g_{a}\in \{a^{=}\},\;g_{b}\in \{b^{=}\},\;such\;that\;g_{b}=a_{1}\;or\;g_{a}=b_{1}\},\;(9)$$

*where a*_1_ = *ag _a_a* ≤ ^−^ *a* *and* *b*_1_ = *bg _b_b* ≤ ^−^ *b*.

*Proof.* Note that outer inverse of any element is a regular element in the ring, and if *x* is any regular element in , then *x* ∈ *x* ∩ *x*.

Now consider any *g _ab_* ∈ {( *ab*) ^=^}. As noted earlier in the Lemma 5, we have *g _ab_* = *g _ab_abg _ab_* in the form of *g _b_g _a_*, where *g _b_* = *g _ab_a* ∈ { *b* ^=^} and *g _a_* = *bg _ab_* ∈ { *a* ^=^}. Now,


$$\begin{array}{l} g_{b}\;=\;g_{ab}a\\ \;=\;abg_{ab}a\;\text{because}\;g_{ab}abg_{ab}=g_{ab}\\ \;=\;ag_{a}a=a_{1}\text{.} \end{array}$$

The proof of *g _a_* = *b*_1_ is similar. From the definition of *a*_1_, it is verified that *g _a_* is a generalized inverse of *a*_1_. Further, we get *a*_1_ *g _a_* = *ag _a_* and *g _a_a*_1_ = *g _a_a*, proving that *a*_1_ ≤ ^−^ *a*. *b*_1_ ≤ ^−^ *b* is similarly proved.

Now to prove ( 9) and the theorem, consider *g _a_* ∈ { *a* ^=^} and *g _b_* ∈ { *b* ^=^} satisfying *g _b_* = *a*_1_ or *g _a_* = *b*_1_ . Now consider the case of *g _b_* = *a*_1_, whereas the other case similar. Since *g _b_* and *a*_1_ are regular elements, we have *g _b_*, *a*_1_ ∈ *g _b_* = *a*_1_ and therefore, *a*_1_ *bg _b_* = *a*_1_ and *g _a_abg _b_* = *g _a_a*. Now, using the same we get


$$(g_{b}g_{a})(ab)(g_{b}g_{a})=g_{b}(g_{a}abg_{b})g_{a}=g_{b}(g_{a}ag_{a})=g_{b}g_{a},$$

proving that *g _b_g _a_* ∈ {( *ab*) ^=^}.

**Remark 1.** *Note that the set* { *g _b_g _a_* : *g _a_* ∈ { *a* ^=^}, *g _b_* ∈ { *b* ^=^}, *such that g _b_* = *a*_1_ *or g _a_* = *b*_1_ } *does not determine exact class of pairs of outer inverses* ( *g _a_*, *g _b_*) *of a and b whose product determine* {( *ab*) ^=^}. *It may be noted in each of the cases (i) g _a_* ⊂ *b*_1_ *(ii) b*_1_ *⊂ g _a_ (iii) a*_1_ *⊂ g _b_ (iv) g _b_ ⊂ a*_1_, *we get*


$$g_{b}g_{a}abg_{b}g_{a}=g_{b}g_{a}\text{.}$$

*Therefore, determining the exact class of* ( *g _a_*, *g _b_*) *determining* {( *ab*) ^=^} *remains to be a problem to probe.*

Let be an associative ring with involution ‘*’. Then, we have the following corollary.

**Corollary 7.** *Given elements a*, *b* ∈ , *if g _a_ and g _b_ are reflexive g-inverses of a and b*, *respectively*, *satisfying any of the cases (a) g _a_* ⊂ *b (b) b* ⊂ *g _a_ (c) a* ⊂ *g _b_ (d) g _b_* ⊂ *a*, *then* $g_{b}g_{a}\in \{{\left(ab\right)}_{r}^{-}\}$ . *Further, we have the following.*

*(i)*  *In the case of (a) or (c) and if g _a_ is a* {1, 2, 3}- *inverse of a, then we have*
$$g_{b}g_{a}\in \{{\left(ab\right)}^{\left\{1,2,3\right\}}\}.$$
*(ii)*  *In the case of (b) or (d) and if g _b_ is a* {1, 2, 4}- *inverse of b, then we have*
$$g_{b}g_{a}\in \{{\left(ab\right)}^{\left\{1,2,4\right\}}\}.$$
*(iii)*  *If a* ^†^, *b* ^†^ exists and a* ⊂ *b*, *then we have*
$$b^{†}a^{†}\in \{{\left(ab\right)}^{\left\{1,2,3\right\}}\}.$$
*(iv)*  *If a* ^†^, *b* ^†^ *exists and b* ⊂ a* , *then we have*
$$b^{†}a^{†}\in \{{\left(ab\right)}^{\left\{1,2,4\right\}}\}.$$
*(v)*  *If a* ^†^, *b* ^†^ *exist and* $a^{*}\underset{cs}{≃}b$ , *then* ( *ab*) ^†^ *exists, in which case*
$${\left(ab\right)}^{†}=b^{†}a^{†}.$$

*Proof.* In the cases of (a), (b), (c), and (d) we have *bg _b_g _a_* = *g _a_*, *g _a_ab* = *b*, *abg _b_* = *a*, and *g _b_g _a_a* = *g _b_*, respectively. Now, by direct substitution it is easily verified that *g _b_g _a_* is a reflexive generalized inverse of *ab*.

If *g _a_* ∈ *a* ^{1,2,3}^ and the condition (a) or (c) is satisfied, then *abg _b_g _a_* = *ag _a_* proving that *g _b_g _a_* ∈ {( *ab*) ^{1,2,3}^}. Hence, (i) is proved.

Similarly, if *g _b_* ∈ *a* ^{1,2,4}^ and the condition (b) or (d) is satisfied, then we have *g _b_g _a_ab* = *g _b_b* proving *g _b_g _a_* ∈ {( *ab*) ^{1,2,4}^} and (ii).

Proof of (iii) and (iv) are consequences of (i) and (ii), respectively, since $a^{†}\underset{sp}{≃}a^{*}$ .

Proof of (v) is an immediate consequence of (iii) and (iv).

Let *A* and *B* be any two complex matrices with *AB* defined. According to Corollary 7 (v), we have ( *AB*) ^†^ = *B* ^†^ *A* ^†^ if *ℛ*( *A*) = *ℛ*( B*), where *ℛ*( *X* ) denotes the row space of *X*. However, the converse need not be true. For example, if


$$A=\left[\begin{array}{l} \begin{array}{l} 1\\ 0 \end{array} \end{array}\;\begin{array}{l} \begin{array}{l} 0\\ 0 \end{array} \end{array}\right]\;\text{and}\;B=\left[\begin{array}{l} \begin{array}{l} 0\\ 1 \end{array} \end{array}\;\begin{array}{l} \begin{array}{l} 1\\ 0 \end{array} \end{array}\right],$$

then ${\left(AB\right)}^{†}=\left[\begin{array}{l} \begin{array}{l} 0\\ 1 \end{array} \end{array}\;\begin{array}{l} \begin{array}{l} 0\\ 0 \end{array} \end{array}\right]$ = *B* ^†^ *A* ^†^. But *ℛ*( B*) ≠ *ℛ*( *A*).

## Left-star and right-star orders

In the present section, we extend the notions of left-star and right-star orders to the case of elements from an associative ring with involution. These notions were originally defined in the matrix case by Baksalary and Mitra in 12. As noted earlier, Drazin proved that the star order is a partial order on a proper *-semigroup and hence on a proper *-ring (see 11).

Following the matrix case, we shall now define the left-star and right-star orders in the context of associative rings with involution.

**Definition 8.** *For a*, *b* ∈ , *we say that a is dominated by b under left-star order*, *and denote a* $\leq_{l}^{*}$ b if a* *a* = a* *b*, *a* ∈ *a and a* ⊆ *b*. *We say that a is dominated by b under right-star order, and denote a* $\leq_{r}^{*}$ b if aa* = ba*, *a* ∈ *a and a* ⊆ *b*.

Baksalary and Mitra in 12 showed that the right-star and left-star orders are partial orders on the class of matrices over complex field. In the case of rings, clearly these relations are reflexive. Further, if *a* $\leq_{l}^{*}$ *b* and *b* $\leq_{l}^{*}$ *c*, then we have a* *a* = a* *b* = a* *c*, since a* = xb* for some *x* and b* *b* = b* *c*. Thus, the the relation $\leq_{l}^{*}$ is transitive and preorder on ring . Additionally, if *a* or *b* is left *-cancellable and *a* $\leq_{l}^{*}$ *b* and *b* $\leq_{l}^{*}$ *a*, then *a* = *b*. For the proof, consider the case of *a* being left *-cacellable. Then, a* *a* = a* *b* ⇒ *a* = *b*, since *b* = *ax* for some *x* and *a* ∈ *a*. Hence, we have the following theorem.

**Theorem 9.** Let be an associative ring with involution ‘* *’*. *Then the relation* $\leq_{l}^{*}$ *(* $\leq_{r}^{*}$ *)* *is a partial order on the class of left* *- *cancellable* ( *right* *- *cancellable*) *elements*. *In particular, if is a proper* *- *ring, then* $\leq_{l}^{*}$ *and* $\leq_{r}^{*}$ *are partial orders on *.

Now, we shall proceed to prove some of the basic properties of left-star ordering. Analogous results for the right-star order can be obtained in the similar manner.

**Theorem 10.** *Let be an associative ring and let a* ∈ *be left* *- *cancellable*. *Then the following statements hold.*

*(i)*  *a* $\leq_{l}^{*}$ *b* ⇔ a* $\leq_{r}^{*}$ b*
*(ii)*  *If p is a projector and a* $\leq_{l}^{*}$ *p*, *then a is also a projector*.
*(iii)*  *If p and q are projectors, then the following are equivalent*.
*(a)*  *p* $\leq_{l}^{*}$ *q*
*(b)*  *p* ⊆ *q*
*(c)*  *p* ⊆ *q*
*(d)*  *p* $\leq_{r}^{*}$ *q*
*(e)*  *p* ≤* *q*
*(f)*  *p* ≤ ^−^ *q*
*(iv)*  *If q is a projector, then a* $\leq_{l}^{*}$ *q* ⇔ *a* $\leq_{r}^{*}$ *q* ⇔ *a* ≤* *q* ⇔ *a* ≤ ^−^ *q*.

*Proof.* Note that (i) follows from the properties of involution ‘*’.

From the hypothesis of (ii), we have a* *a* = a* *p* = *pa* and *a* ∈ *a* ⊆ *p*. Therefore, *a* = *pa* = a* *a* proving that *a* is a projector. This proves (ii).

The equivalences of (a)-(f) in (iii) are easily verified using the properties of projectors.

Proof of (iv) follows from the statements (ii) and (iii).

Now, for *a*, *b*, *c* ∈ ^†^, we make the following observations.

(i)  *a* $\leq_{l}^{*}$ *b* ⇔ *a* ^†^ $\leq_{l}^{*}$ *b* ^†^ and *a* $\leq_{r}^{*}$ *b* ⇔ *a* ^†^ $\leq_{r}^{*}$ *b* ^†^: If *a* $\leq_{l}^{*}$ *b*, then a* *a* = a* *b*, *a* ∈ *a* and *a* ⊆ *b*. So, a* *a* = b* *a* and *a* ^†^ = a* ⊆ b* = *b* ^†^. Then, we have *bb* ^†^ *a* = *a* and *a* ^†^ *bb* ^†^ = *a* ^†^. Thus, *a* ^†^ ∈ . Also, *a* ^†^ *a* = *a* ^†^ *b* = b*( *a* ^†^)* and hence,
$$a^{†}\subseteq b^{*}=b^{†}\;(10)$$
  Further,
$$(a^{†}{)}^{*}b^{†}=(a^{†}{)}^{*}a^{†}ab^{†}=(a^{†}{)}^{*}a^{†}bb^{†}=(a^{†}{)}^{*}a^{†}\;(11)$$
  Thus, *a* ^†^ $\leq_{l}^{*}$ *b* ^†^. Converse part follows by the uniqueness of Moore-Penrose inverse and the fact that ( *a* ^†^) ^†^ = *a*. A similar argument will prove the second equivalence.
(ii)  *a* $\leq_{l}^{*}$ *b* ⇔ *aa* ^†^ = *ab* ^†^ ( *a* $\leq_{r}^{*}$ *b* ⇔ *a* ^†^ *a* = *b* ^†^ *a*): Follows by noting that *aa* ^†^ = *ab* ^†^ is equivalent to ( 10) and ( 11).
(iii)  If *a* $\leq_{l}^{*}$ *b*, then *b* ^†^ is a {1,3}-inverse of *a* (If *a* $\leq_{r}^{*}$ *b*, then *b* ^†^ is a {1,4}-inverse of *a*): From (ii) above, it follows that *ab* ^†^ is Hermitian. Also, postmultiplying *aa* ^†^ = *ab* ^†^ by *a*, we get that *b* ^†^ is a g-inverse of *a*.
(iv)  If *a* $\leq_{l}^{*}$ *b* (or *a* $\leq_{r}^{*}$ *b*), then ( *bb* ^†^ *a*) ^†^ = *a* ^†^ *bb* ^†^ and ( *ab* ^†^ *b*) ^†^ = *b* ^†^ *ba* ^†^: If *a* $\leq_{l}^{*}$ *b*, then we have *a* ⊆ *b*, *a* ^†^ ⊆ *b* ^†^, *a* ^†^ ⊆ *b* ^†^ (refer (i) above) and hence, *a* ⊆ *b*. From the first two inclusions, we get that *bb* ^†^ *a* = *a* and *a* ^†^ *bb* ^†^ = *a* ^†^ proving that ( *bb* ^†^ *a*) ^†^ = *a* ^†^ *bb* ^†^. The last two inclusions imply the equations *b* ^†^ *ba* ^†^ = *a* ^†^ and *ab* ^†^ *b* = *a* proving that ( *ab* ^†^ *b*) ^†^ = *b* ^†^ *ba* ^†^.
(v) 
$\leq_{l}^{*}$ and $\leq_{r}^{*}$ are partial orders on ^†^: Evidently, $\leq_{l}^{*}$ is reflexive. Suppose that *a* $\leq_{l}^{*}$ *b* and *b* $\leq_{l}^{*}$ *c*. Then *a* = ( *a* ^†^)* a* *a* = ( *a* ^†^)* a* *b* = *aa* ^†^ *b* = *b* as it follows from *b* ⊆ *a* that *aa* ^†^ *b* = *b*. Hence the anti-symmetry. Now for some *a*. *b*, *c* ∈ , suppose that *a* $\leq_{l}^{*}$ *b* and *b* $\leq_{l}^{*}$ *c*. Then a* *a* = a* *b* and b* *b* = b* *c* with *a* ⊆ *b* ⊆ *c*. Now,
$$a^{*}a=a^{*}b=a^{*}(b^{†}{)}^{*}b^{*}b=a^{*}(b^{†}{)}^{*}b^{*}c=(bb^{†}a{)}^{*}c=a^{*}c$$
  as it follows from *a* ⊆ *b* that *bb* ^†^ *a* = *a*. Hence, $\leq_{l}^{*}$ is transitive, which proves that $\leq_{l}^{*}$ is a partial order.
(vi)  The minus order dominates the left-star and right-star orders: Suppose that *a* $\leq_{l}^{*}$ *b*. Then *a* ^†^ *a* = *a* ^†^ *b* and *a* ⊆ *b*. So, *a* = *aa* ^†^ *b* and *a* = *bb* ^−^ *a* for any g-inverse *b* ^−^ of *b*. Hence, *a* ∈ *b* ∩ *b*. Also,
$$ab^{-}a=aa^{†}ab^{-}a=aa^{†}bb^{-}a=aa^{†}a=a$$
  and hence { *b* ^−^} ⊆ { *a* ^−^}. Now, from Theorem 2 it follows that *a* ≤ ^−^ *b*.
(vii)  If *a* ≤* *b*, then *b* ^†^ is a {1,3,4}-inverse of *a* and *b* ^†^ *ab* ^†^ = *a* ^†^: The proof that *b* ^†^ is a {1,3,4}-inverse of *a* follows from the equivalence given in ( 6). Further, using the same equivalence, we get that *b* ^†^ *ab* ^†^ = *b* ^†^ *aa* ^†^ = *a* ^†^ *aa* ^†^ = *a* ^†^.
  From ( 7), we have *a* ^†^ ≤* *b* ^†^. In fact, if *g* is a {1,3,4}-inverse of *a*, then
$$(a^{†}{)}^{*}a^{†}=(a^{†}{)}^{*}a^{†}(a^{†}{)}^{*}a^{*}g^{*}a^{*}=(a^{†}{)}^{*}a^{†}g^{*}a^{*}=(a^{†}{)}^{*}a^{†}ag=(a^{†}{)}^{*}g$$
  and similarly, *a* ^†^( *a* ^†^)* = *g*( *a* ^†^)*. Hence, *a* ^†^ ≤* *g*. Therefore, we have
$$a^{†}=\min\{g:aga=a,{\left(ag\right)}^{*}=ag,{\left(ga\right)}^{*}=ga\},$$
  where the minimum is with respect to the star order (see Theorem 2 ^11^).
(viii) If *c* ≤* *a*, then *c* ^†^ is a {2,3,4}-inverse of *a*: The proof follows by ( 5). From ( 7), we have *c* ^†^ ≤* *a* ^†^. In fact, it is true that *h* ≤* *a* ^†^ for any {2,3,4}-inverse *h* of *a*. Thus, we have
$$a^{†}=\max\{h\;:\;hah=h,{\left(ah\right)}^{*}=ah,{\left(ha\right)}^{*}=ha\},$$
  where the maximum is with respect to the star order ( Theorem 2 ^11^).
(ix) The following implication follows from ( 5),
$$a\leq^{*}b\Rightarrow a\leq^{-}b.\;(12)$$
  If *a* ≤* *b* and *c* = *b* − *a*, then by ( 12) and Theorem 2 it follows that *b* = *a* ⊕ *c*. Moreover, *b* ^†^ exists if and only if *a* ^†^ and *c* ^†^ exist, in which case,
$$b^{†}=a^{†}⊕c^{†}\;(13)$$
  This can be seen as follows: If *a* ≤* *b*, then a* *c* = a* *b* − a* *a* = 0, which implies *a* ^†^ *c* = 0. Similarly, *ca* ^†^ = 0, *ac* ^†^ = 0, and *c* ^†^ *a* = 0.

## The reverse order law for Moore-Penrose inverse

The projectors play a prominent role in obtaining several equivalent conditions for the reverse order law for Moore-Penrose inverse. They have distinct properties associated with the star order, a few of which are discussed in 29. In this section, we shall note a few more interesting behaviors of projectors associated with star, left-star and right-star orders. An important observation in proving our main theorem is the following fact: For a projector *p*, we have *pa* $\leq_{r}^{*}$ *a* ( *ap* $\leq_{l}^{*}$ *a*) if and only if *pa* ≤* *a* ( *ap* ≤* *a*).

**Lemma 11.** *Let be an associative ring with involution* ‘*’ and let *p* be a projector. For any *a* ∈ , the following are equivalent.

(i) ( *pa*)( *pa*)* = *a*( *pa*)*
(ii) paa* ∈ *^o^* ( *p* − 1)
(iii) *p* commutes with aa*
(iv) paa* is Hermitian
(v) *pa* ≤* *a*

In particular, if *a* ∈ *a*, then


$$pa\leq_{r}^{*}a\;\text{if}\;\text{and}\;\text{only}\;\text{if}\;pa\leq^{*}a.\;(14)$$

*Proof.* Note that (i)⇒(ii), (ii)⇒(iii), and (iii)⇒(iv) are obvious.

Evidently, ( *pa*)*( *pa*) = ( *pa*)* *a*. Now, *pa*( *pa*)* = *p*( *pa*) a* = paa* = *a*( *pa*)*. This proves (iv)⇒(v).

Now, ( 14) follows from the definition of star order, equivalence of (i) and (v), and by noting that *a* ∈ *a* implies that *pa* ∈ *pa*, as *p* is idempotent.

Symmetrically, we have the following result.

**Lemma 12.** *Let be an associative ring with involution* ‘*’ and let *p* be a projector. For any *a* ∈ , the following are equivalent.

(i) ( *ap*)*( *ap*) = ( *ap*)* *a*
(ii) a* *ap* ∈ (1 − *p*) ^*o*^
(iii) *p* commutes with a* *a*
(iv) a* *ap* is Hermitian
(v) *ap* ≤* *a*

In particular, if *a* ∈ *a*, then


$$ap\leq_{l}^{*}a\;\text{if}\;\text{and}\;\text{only}\;\text{if}\;ap\leq^{*}a.\;(15)$$

**Lemma 13.** *Let be an associative ring with involution* ‘*’ and let *a* ∈ . If *p* is a projector commuting with *a*, then *pa* ≤* *a* and *ap* ≤* *a*.

*Proof.* Follows by noting that the hypothesis implies statement (iii) of Lemma 11 as well as Lemma 12.

Now we prove the main theorem of the present paper. The equivalence of statements (i) and (vii) is discussed in 2 and in 21 and the equivalence of (i) and (vi) is proved in 30. However, our proof is in the general setting of Lemma 11 and Lemma 12, which use the properties of projectors.

**Theorem 14.** *Let be a proper* *- *ring. For a*, *b* ∈ ^†^, *the following are equivalent.*

(i) ( *ab*) ^†^ = *b* ^†^ *a* ^†^
(ii) ( *a* ^†^ *ab*) ^†^ = *b* ^†^ *a* ^†^ *a and* ( *abb* ^†^) ^†^ = *bb* ^†^ *a* ^†^.
(iii) *a* ^†^ *ab* $\leq_{r}^{*}$ *b and bb* ^†^ $\leq_{l}^{*}$ *a*
(iv) ( *a* ^†^ *ab*)( *a* ^†^ *ab*)* = *b*( *a* ^†^ *ab*)* *and* ( *abb* ^†^)*( *abb* ^†^) = ( *abb* ^†^)* *a*
(v) *a* ^†^ abb* ∈ *^o^* ( *a* ^†^ *a* − 1) and a* *abb* ^†^ ∈ (1 − *bb* ^†^) *^o^*
(vi) *a* ^†^ abb* = bb* *a* ^†^ a and a* *abb* ^†^ = *bb* ^†^ a* *a*
(vii) *a* ^†^ abb* and a* *abb* ^†^ *are Hermitian*
(viii) *a* ^†^ *ab* ≤* *b* *and* *abb* ^†^ ≤* *a*

*Proof.* (i)⇒(ii) can be verified by direct substitution. Conversely, premultiplying *a* ^†^ *ab*( *b* ^†^ *a* ^†^ *a*) *a* ^†^ *ab* = *a* ^†^ *ab* by *a* and postmultiplying ( *b* ^†^ *a* ^†^ *a*) *a* ^†^ *ab*( *b* ^†^ *a* ^†^ *a*) = *b* ^†^ *a* ^†^ *a* by *a* ^†^, we get *abb* ^†^ *a* ^†^ *ab* = *ab* and *b* ^†^ *a* ^†^ *abb* ^†^ *a* ^†^ = *b* ^†^ *a* ^†^, respectively. Also, we have *abb* ^†^ *a* ^†^ = *abb* ^†^( *bb* ^†^ *a* ^†^) and *b* ^†^ *a* ^†^ *ab* = ( *b* ^†^ *a* ^†^ *a*) *a* ^†^ *ab*. Hence, *abb* ^†^ *a* ^†^ and *b* ^†^ *a* ^†^ *ab* are Hermitian. Thus, we have ( *ab*) ^†^ = *b* ^†^ *a* ^†^, proving (ii)⇒(i). This proves (i)⇔(ii).

The equivalence of the statements (iii)-(viii) will follow from the Lemma 11 and Lemma 12 noting that *a* ^†^ *a* and *bb* ^†^ are projectors. It remains to prove that (i) *⇔*(viii).

(i)⇔(viii): Evidently, ( *a* ^†^ *ab*)* *a* ^†^ *ab* = b* a*( *a* ^†^)* *a* ^†^ *ab* = ( b* *a* ^†^ *a*) *b* = ( *a* ^†^ *ab*)* *b*.

Premultiplying b* a* = *b* ^†^ *a* ^†^ *ab*( *ab*)* by abb* *b*, we get


$$abb^{*}bb^{*}a^{*}=abb^{*}bb^{†}a^{†}ab{\left(ab\right)}^{*}=abb^{*}a^{†}abb^{*}a^{*}.$$

Taking *u* = bb* a* and *v* = *a* ^†^ abb* a*, we note that u* *u* = u* *v* = v* *u* = v* *v* = abb* *a* ^†^ abb* a* and hence *u* = *v*, since ‘*’ is a proper involution. That is, bb* a* = *a* ^†^ abb* a*. Therefore, we get


$$a^{†}ab{\left(a^{†}ab\right)}^{*}=a^{†}abb^{*}a^{*}(a^{†}{)}^{*}=bb^{*}a^{*}(a^{†}{)}^{*}=b{\left(a^{†}ab\right)}^{*}$$

which proves that *a* ^†^ *ab* ≤* *b*. Proof of other inequality is symmetric.

Conversely, we have that *b* ^†^ and *a* ^†^ are {1,3,4}-inverses of *a* ^†^ *ab* and *abb* ^†^, respectively. Hence, *b* ^†^ *a* ^†^ *ab* and *abb* ^†^ *a* ^†^ are Hermitian. Also, premultiplying *a* ^†^ *abb* ^†^ *a* ^†^ *ab* = *a* ^†^ *ab* by *a*, we get *abb* ^†^ *a* ^†^ *ab* = *ab*.

Further, ( *a* ^†^ *ab*)* ⊆ b* = *b* ^†^, gives


$${\left(a^{†}ab\right)}^{*}bb^{†}={\left(a^{†}ab\right)}^{*}\;\text{i.e}.,\;b^{*}a^{†}abb^{†}=b^{*}a^{†}a$$

Premultiplying by *b* ^†^( *b* ^†^)* and post-multiplying by *a* ^†^, we get


$$\;b^{†}a^{†}abb^{†}a^{†}=b^{†}a^{†}$$

proving (i).

From (i)⇔ (ii) of Theorem 14, we have ( *a* ^†^ *ab*) ^†^ = *b* ^†^ *a* ^†^ *a* and ( *abb* ^†^) ^†^ = *bb* ^†^ *a* ^†^ if and only if the reverse order law for Moore-Penrose inverse holds. Further from (i)⇔(viii) and ( 7), we have ( *ab*) ^†^ = *b* ^†^ *a* ^†^ if and only if ( *a* ^†^ *ab*) ^†^ ≤* *b* ^†^ and ( *abb* ^†^) ^†^ ≤* *a* ^†^. Hence, we have the following corollary.

**Corollary 15.** *Let be a proper* *- *ring and let a*, *b* ∈ ^†^. *Then the following are equivalent*.

(i) ( *ab*) ^†^ = *b* ^†^ *a* ^†^
(ii) *b* ^†^ *a* ^†^ *a* $\leq_{l}^{*}$ *b* ^†^ *and bb* ^†^ *a* ^†^ $\leq_{r}^{*}$ *a* ^†^
(iii) ( *b* ^†^ *a* ^†^ *a*)*( *b* ^†^ *a* ^†^ *a*) = ( *b* ^†^ *a* ^†^ *a*)* *b* ^†^ *and* ( *bb* ^†^ *a* ^†^)( *bb* ^†^ *a* ^†^)* = *a* ^†^( *bb* ^†^ *a* ^†^)*
(iv) ( *b* ^†^)* *b* ^†^ *a* ^†^ *a* ∈ ( *a* ^†^ *a* − 1) *^o^ and bb* ^†^ *a* ^†^( *a* ^†^)* ∈ *^o^* (1 − *bb* ^†^)
(v) *a* ^†^ *a*( *b* ^†^)* *b* ^†^ = ( *b* ^†^)* *b* ^†^ *a* ^†^ *a and a* ^†^( *a* ^†^)* *bb* ^†^ = *bb* ^†^ *a* ^†^( *a* ^†^)*
(vi) *a* ^†^ *a*( *b* ^†^)* *b* ^†^ *and a* ^†^( *a* ^†^)* *bb* ^†^ *are Hermitian*
(vii) *b* ^†^ *a* ^†^ *a* ≤* *b* ^†^ *and bb* ^†^ *a* ^†^ ≤* *a* ^†^

From Theorem 14, we have that *a* ^†^ *ab* $\leq_{r}^{*}$ *b* and *abb* ^†^ $\leq_{l}^{*}$ *a* if and only if the reverse order law ( *ab*) ^†^ = *b* ^†^ *a* ^†^ holds. The equivalence fails to exist if we interchange the left-star and right star orders. However, one of the implications is true and the same is proved in the following theorem.

**Theorem 16.** *Let be a proper* *- *ring. Let a*, *b* ∈ *be such that a* ^†^ *and b* ^†^ *exist. Then,*


$${\left(ab\right)}^{†}=b^{†}a^{†}\;\Rightarrow \;\{\begin{array}{l} \begin{array}{l} a^{†}ab\leq_{l}^{*}b\\ abb^{†}\leq_{r}^{*}a. \end{array} \end{array}$$

*Proof.* We have, ( *a* ^†^ *ab*)*( *a* ^†^ *ab*) = b* a*( *a* ^†^)* *a* ^†^ *ab* = b* *a* ^†^ *ab* = ( *a* ^†^ *ab*)* *b*. Further, taking *u* = *a* ^†^ *ab* and *v* = *bb* ^†^ *a* ^†^ *ab*, we get u* *u* = u* *v* = v* *u* = v* *v* = b* *a* ^†^ *ab*.

Thus,


$$a^{†}ab=bb^{†}a^{†}ab\;\text{and}\;\text{hence}\;a^{†}ab\subseteq b$$

which proves that *a* ^†^ *ab* $\leq_{l}^{*}$ *b*. Proof of other inequality is symmetric.

**Remark 2.** *The converse of the above theorem need not be true in general. For example, if*


$$A=\left[\begin{array}{l} \begin{array}{l} 1\\ 0 \end{array} \end{array}\;\begin{array}{l} \begin{array}{l} \frac{1}{2}\\ 0 \end{array} \end{array}\right]\;and\;B=\left[\begin{array}{l} \begin{array}{l} 1\\ 0 \end{array} \end{array}\;\begin{array}{r} \begin{array}{r} -1\\ 2 \end{array} \end{array}\right],$$

*then*


$$A^{†}=\frac{4}{5}\left[\begin{array}{l} \begin{array}{l} 1\\ \frac{1}{2} \end{array} \end{array}\;\begin{array}{l} \begin{array}{l} 0\\ 0 \end{array} \end{array}\right]\;,\;B^{†}=B^{-1}=\frac{1}{2}\left[\begin{array}{l} \begin{array}{l} 2\\ 0 \end{array} \end{array}\;\begin{array}{l} \begin{array}{l} 1\\ 1 \end{array} \end{array}\right]$$

*so that*


$$A^{†}AB=\frac{4}{5}\left[\begin{array}{l} \begin{array}{l} 1\\ \frac{1}{2} \end{array} \end{array}\;\begin{array}{l} \begin{array}{l} 0\\ 0 \end{array} \end{array}\right]\;and\;ABB^{†}=\left[\begin{array}{l} \begin{array}{l} 1\\ 0 \end{array} \end{array}\;\begin{array}{l} \begin{array}{l} \frac{1}{2}\\ 0 \end{array} \end{array}\right].$$

*Clearly, A* ^†^ *AB* $\leq_{l}^{*}$ *B* *and ABB* ^†^ $\leq_{r}^{*}$ *A. But*


$${\left(AB\right)}^{†}=\left[\begin{array}{l} \begin{array}{l} 1\\ 0 \end{array} \end{array}\;\begin{array}{l} \begin{array}{l} 0\\ 0 \end{array} \end{array}\right]\neq \left[\begin{array}{l} \begin{array}{l} 1\\ \frac{1}{5} \end{array} \end{array}\;\begin{array}{l} \begin{array}{l} 0\\ 0 \end{array} \end{array}\right]=B^{†}A^{†}.$$

From Lemma 11 and Lemma 12, we have that *a* ^†^ *ab* $\leq_{r}^{*}$ *b* implies *a* ^†^ *ab* ≤* *b* and *abb* ^†^ $\leq_{l}^{*}$ *a* implies *abb* ^†^ ≤* *a*. It is not true in general that *a* ^†^ *ab* ≤* *b* whenever *a* ^†^ *ab* $\leq_{l}^{*}$ *b* and that *abb* ^†^ ≤* *a* whenever *abb* ^†^ $\leq_{r}^{*}$ *a*. The following lemma gives a sufficient condition for this result to hold.

**Theorem 17.** *Let be a proper* *- *ring and let a*, *b* ∈ ^†^ *. Suppose* ( *ab*) ^†^ = *b* ^†^ *a* ^†^ *. Then, a* ^†^ *ab* $\leq_{l}^{*}$ *b implies a* ^†^ *ab* ≤* *b* *and abb* ^†^ $\leq_{r}^{*}$ *a implies abb* ^†^ ≤* *a.*

*Proof.* Since *a* ^†^ *ab* ⊆ *b* , we have


$$\begin{array}{l} a^{†}ab(a^{†}ab{)}^{*}=bb^{†}a^{†}ab(a^{†}ab{)}^{*}\\ \;=bb^{†}a^{†}ab(ab{)}^{*}(a^{†}{)}^{*}\\ \;=b(ab{)}^{*}((ab{)}^{†}{)}^{*}(ab{)}^{*}(a^{†}{)}^{*}\\ \;=b(ab{)}^{*}(a^{†}{)}^{*}\\ \;=b(a^{†}ab{)}^{*} \end{array}$$

as desired. The second implication can be proved similarly.

The proof of the following theorem is immediate from Theorem 14 and Lemma 13, noting that *a* ^†^ *a* and *bb* ^†^ are projectors commuting with *b* and *a*, respectively. This theorem shows that only two commutative relations out of four, given by Erdelyi in (Theorem 1 ^1^) are sufficient for the reverse order law.

**Theorem 18.** *Let be a proper* *- *ring and let a*, *b* ∈ *be such that a* ^†^ *and b* ^†^ *exist. If both a*( *bb* ^†^) = ( *bb* ^†^) *a and b*( *a* ^†^ *a*) = ( *a* ^†^ *a*) *b are true, then* ( *ab*) ^†^ = *b* ^†^ *a* ^†^.

## Conclusions

In the present paper, the reverse order law for outer inverses is discussed. The notions of left-star and right-star orderings are extended to the case of associative rings with involution and several properties of the same are discussed. Also, necessary and sufficient conditions—in terms of star, left-star and right-star orders—for the reverse order law for Moore-Penrose inverse are given. The discussion on left-star and right-star partial orderings appears to help in further study of relation between star partial order and column space decomposition of matrices. Further, it has been noted in Theorem 10 that, in the class of all projectors the star, left-star, right-star and minus orders are all equivalent. The classification of elements of associative rings for which any two or more orders are equivalent seems to be an interesting problem to explore and the same will be presented in the subsequent papers.

## Data availability

### Underlying data

All data underlying the results are available as part of the article and no additional source data are required.
