# Supplementary material for: Reverse order law for outer inverses and Moore-Penrose inverse in the context of star order
Source: F1000Res. 2022 Jul 27;11:843. [Version 1] doi: 10.12688/f1000research.123411.1 (PMC10130701; doi:10.12688/f1000research.123411.1)
Supplement: Peer review history

# Peer review: Reviewer response for version 1

**DOI:** 10.5256/f1000research.135513.r169472

**Published:** 2023-04-25

**Authors:** Mehsin Jabel Atteya

**Affiliations:** 1 Department of Mathematics, Al- Mustansiriyah University, Falastin St, Baghdad, Iraq

At the beginning, the counter examples and examples which are used in this article assist in understanding the specific points. During this paper, the authors provide background information about this subject in the introduction with preliminary results which are used to discuss the rest of the developments.This information is written in the first two sections. While the characterized the class of pairs of outer inverses which satisfy the extended reverse order law. Additionally, interestingly, the theory of minus partial order plays an important role in obtaining this characterization, in the third section. In the fourth and fifth sections, the authors posted necessary and sufficient relations on the elements for the Moore-Penrose inverses of those elements satisfying reverse order law. Also, they introduce and study left-star partial order and right-star partial order for the elements from associative ring. Finally, all the results which harvested in this paper are mathematically correct and interesting for more researchers.

  strongly recommend this paper for publishing.

Is the work clearly and accurately presented and does it cite the current literature?

Yes

If applicable, is the statistical analysis and its interpretation appropriate?

Yes

Are all the source data underlying the results available to ensure full reproducibility?

Yes

Is the study design appropriate and is the work technically sound?

Yes

Are the conclusions drawn adequately supported by the results?

Yes

Are sufficient details of methods and analysis provided to allow replication by others?

Yes

Reviewer Expertise:

Algebra, Quantum Group, Ring Theory.

I confirm that I have read this submission and believe that I have an appropriate level of expertise to confirm that it is of an acceptable scientific standard.

# Peer review: Reviewer response for version 1

**DOI:** 10.5256/f1000research.135513.r146946

**Published:** 2022-10-21

**Authors:** Balaji Ramamurthy

**Affiliations:** 1 Department of Mathematics, Indian Institute of Technology Madras, Chennai, Tamil Nadu, India

The reverse order law in the case of matrix inverses i.e., (AB) ^−1^ = B ^−1^A ^−1^ is well known in the literature. But the same is not true when the inverses are replaced by generalized inverses. However, in the case of rectangular matrices the reverse order law could be extended by replacing the inverses by Moore-Penrose inverse subject to some necessary and sufficient conditions. When we consider the elements over an associative ring many tools which are available in the context of matrices, like rank, column space and row space are missing and hence the study of reverse order law for non-invertible elements is an interesting problem.

 In the present paper, the authors study the reverse order law extended for outer inverses and Moore-Penrose inverses of the elements from associative ring using the minus partial order and the star order. These techniques are quite innovative and interesting.

 In the first two sections authors provide detailed introduction and preliminary results required to discuss the rest of the developments.

 In the third section the authors characterized the class of pairs of outer inverses satisfying the extended reverse order law. Interestingly, the theory of minus partial order plays an important role in obtaining this characterization.

 In the fourth section the authors introduce and study left-star partial order and right-star partial order for the elements from associative ring. The interesting properties of these partial orders are effectively use in the Section 5 to obtain necessary and sufficient conditions on the elements for the Moore-Penrose inverses of those elements satisfying reverse order law.

 All the results obtained in this paper are mathematically correct and interesting. The examples and counter examples discussed in this paper help in understanding the subject. I strongly recommend this paper for indexing.

Is the work clearly and accurately presented and does it cite the current literature?

Yes

If applicable, is the statistical analysis and its interpretation appropriate?

Yes

Are all the source data underlying the results available to ensure full reproducibility?

Yes

Is the study design appropriate and is the work technically sound?

Yes

Are the conclusions drawn adequately supported by the results?

Yes

Are sufficient details of methods and analysis provided to allow replication by others?

Yes

Reviewer Expertise:

Linear algebra

I confirm that I have read this submission and believe that I have an appropriate level of expertise to confirm that it is of an acceptable scientific standard.
